# Immune cells as messengers from the CNS to the periphery: the role of the meningeal lymphatic system in immune cell migration from the CNS

**DOI:** 10.3389/fimmu.2023.1233908

**Published:** 2023-08-17

**Authors:** Collin Laaker, Cameron Baenen, Kristóf G. Kovács, Matyas Sandor, Zsuzsanna Fabry

**Affiliations:** ^1^ Neuroscience Training Program, University of Wisconsin Madison, Madison, WI, United States; ^2^ Department of Pathology and Laboratory Medicine, School of Medicine and Public Health, University of Wisconsin Madison, Madison, WI, United States

**Keywords:** meningeal lymphatics, immune cell migration, dendritic cells, cribriform plate, cervical lymph node, olfactory nerves, CNS

## Abstract

In recent decades there has been a large focus on understanding the mechanisms of peripheral immune cell infiltration into the central nervous system (CNS) in neuroinflammatory diseases. This intense research led to several immunomodulatory therapies to attempt to regulate immune cell infiltration at the blood brain barrier (BBB), the choroid plexus (ChP) epithelium, and the glial barrier. The fate of these infiltrating immune cells depends on both the neuroinflammatory environment and their type-specific interactions with innate cells of the CNS. Although the fate of the majority of tissue infiltrating immune cells is death, a percentage of these cells could become tissue resident immune cells. Additionally, key populations of immune cells can possess the ability to “drain” out of the CNS and act as messengers reporting signals from the CNS toward peripheral lymphatics. Recent data supports that the meningeal lymphatic system is involved not just in fluid homeostatic functions in the CNS but also in facilitating immune cell migration, most notably dendritic cell migration from the CNS to the meningeal borders and to the draining cervical lymph nodes. Similar to the peripheral sites, draining immune cells from the CNS during neuroinflammation have the potential to coordinate immunity in the lymph nodes and thus influence disease. Here in this review, we will evaluate evidence of immune cell drainage from the brain via the meningeal lymphatics and establish the importance of this in animal models and humans. We will discuss how targeting immune cells at sites like the meningeal lymphatics could provide a new mechanism to better provide treatment for a variety of neurological conditions.

## 1 Introduction

The brain is a highly specialized organ that is protected by its unique system of barriers, which regulate its internal immunity. Classically considered an “immune privileged” organ due to its tightly restricted trafficking of peripheral immune cells into the brain parenchyma during steady-state conditions, the label of immune privilege should not be mistaken for an absence of immune cells in the CNS. Within the CNS there are populations of resident microglia and diverse populations of peripheral-derived leukocytes that patrol various non-parenchymal compartments surrounding the brain in the meninges, perivascular spaces, and in the choroid plexus (1, 2). However, during inflammatory states, peripherally-derived immune cell populations can access the brain tissue and surrounding meningeal layers of the CNS, migrating across barrier sites in substantial numbers. Diseases like Multiple Sclerosis (MS), Stroke, Alzheimer’s disease, and brain cancer, are all associated with infiltrating immune cells that can directly influence disease progression. In ischemic stroke for example, predictable waves of neutrophils, T cells, and monocytes can be found entering the brain in the hours and days after a stroke has occurred (3, 4). These CNS-infiltrating immune cells can then exacerbate or alleviate the damaged ischemic brain in the resulting immune response (5). In MS, the core pathology of the disease is more directly caused by infiltrating immune cells, namely autoreactive immune cells which migrate to the brain and spinal cord to target myelin. Thus, for many years immense research has been focused on understanding the cell to cell interactions that mediate the infiltration of immune cells across the blood brain barrier in the context of MS. In essence, this paradigm laid the groundwork for numerous clinical trials with the underlying goal to target or block immune cells in the periphery before they can infiltrate past the BBB and cause damage. This line of research yielded therapies like Natalizumab, a drug that blocks adhesion and trafficking of leukocytes across blood brain barrier endothelial cells, which has been used for nearly two decades to treat MS (6).

While this model of understanding neuroinflammation through the lens of immune cell infiltration into the CNS has proven reliable and generated useful therapies across a wide range of diseases, often overlooked is the process of immune cell migration out of the CNS and its associated structures. Indeed, while immune cells that infiltrate into the brain are generally on a one-way ticket to carry out their effector functions and die, there are exceptions. Just as seen with infiltration into the brain, migration of immune cells from the brain into the periphery can have important pathological consequences and therapeutic potential. Recent studies have demonstrated that retention of immune cells within the brain and disruption of normal immune cell migration mechanisms from the CNS can influence pathology, and that specific populations of CNS-migrating immune cells, particularly dendritic cells (DCs), can coordinating immunity through the delivery of antigen to lymph nodes (7–10). During multiple sclerosis for example, myelin and neuronal antigen are found within the APCs of the cervical lymph nodes of MS patients, with the amount correlating with disease severity (11). Supporting this, surgical resection of CNS-draining lymph nodes, resulted in reduced EAE severity in rodent models (12, 13), implicating the pathological relevance of CNS antigen drainage. Additionally, recent characterizations of the meningeal lymphatic vessels which surround the brain have provided a clearer picture of exactly how fluid, waste, antigen, and immune cells may be trafficked out of the CNS toward the cervical lymph nodes (14). Here in this review, we will provide a broad overview of the literature regarding the evidence of immune cell migration out of the CNS from the perspective of several immune cell populations involved, and their relationship to meningeal lymphatic drainage pathways. For each immune cell we will also put into context the importance of this migration during neuroinflammation and treatment of neurological diseases. Finally, it should be noted that immune cells represent only a single component of the CNS’s drainage repertoire. Drainage of soluble antigens, waste, and fluid are also important and are hypothesized to share similar pathways of drainage as immune cells. However, the purpose of this review is to focus on immune cells themselves which migrate through the meningeal lymphatic system, not the full dynamics of the system (waste, fluid, soluble antigen, etc). For a more holistic review of the pathways and mechanics of cerebrospinal fluid (CSF) movement and solute drainage from the CNS see the following reviews (15–17).

## 2 Clearance routes from the CNS and meninges

**Figure 1:**
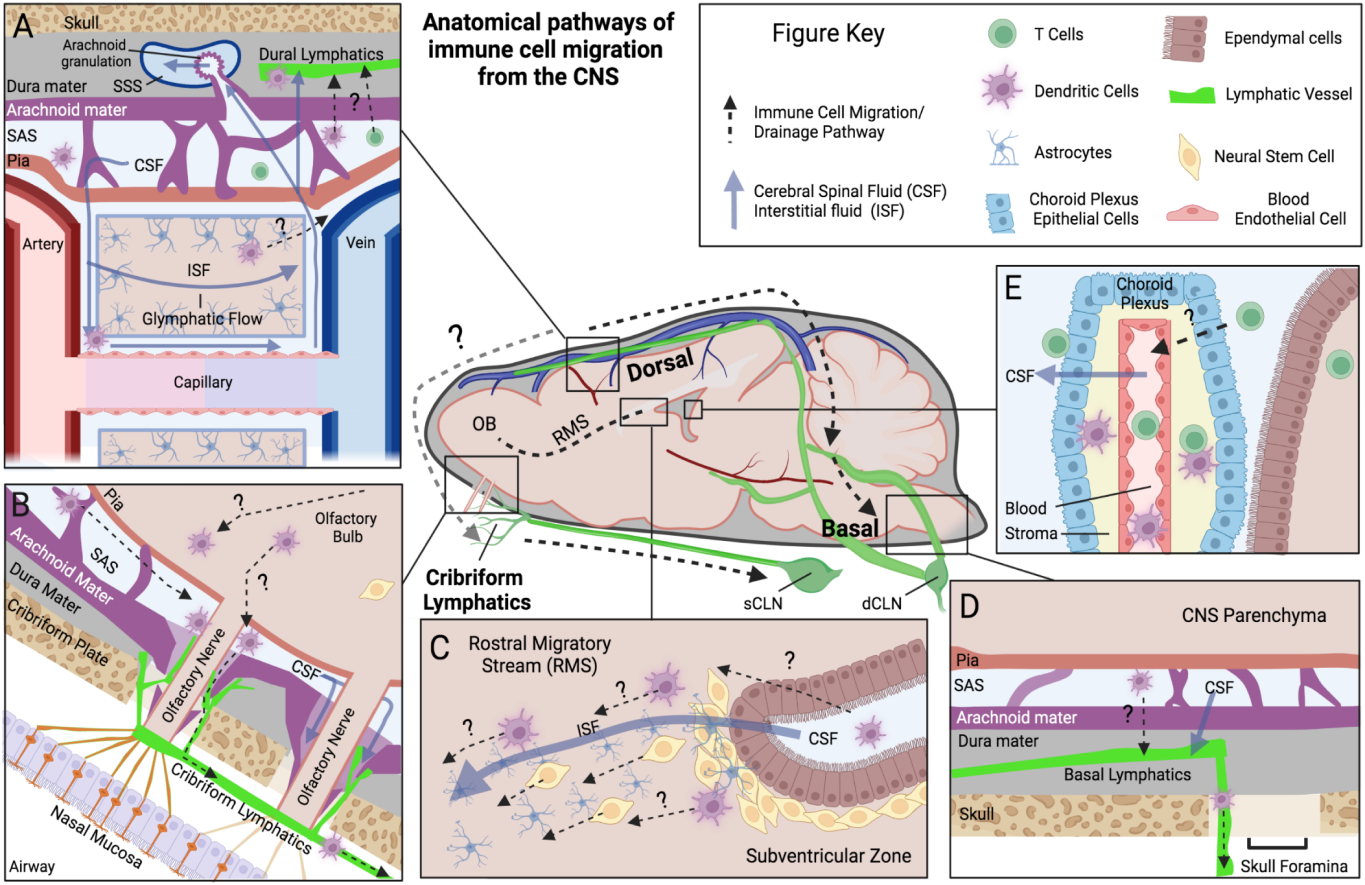
Anatomical pathways of immune cell migration from the CNS. Schematic outlining the anatomical locations of a few of the proposed pathways of immune cell migration (Dotted lines) within the brain and meningeal compartments, derived from rodent animal experiments. Common fluid movement pathways within CNS are also outlined (Blue solid lines). Each panel represents a unique anatomical site of proposed immune cell migration: **(A)** Dorsal dural lymphatics and Glymphatic system. Here perivascular CSF glymphatic flow is hypothesized to drive fluid transport across parenchymal space and into SAS. Immune cells are thought to utilize these perivascular pathways to reach dorsal leptomeningeal spaces (Pia, SAS, and Arachnoid). Once in the SAS, immune cells are proposed to cross the arachnoid mater and traffic to lymphatic vessels in the dural layer, potentially at “hotspots” where arachnoid barriers are more permissible such as arachnoid granulations. Evidence for this is limited however, with arachnoid granulations being primarily a human phenomenon and their relationship mostly investigated for fluid/solute flow into the superior sagittal sinus, not immune cells. **(B)** Olfactory Bulb-Cribriform Lymphatic axis. Olfactory nerves protrude out of the olfactory bulb through skull foramina at the cribriform plate. Along these nerves the meningeal layers have been demonstrated to be discontinuous, with potential gaps in arachnoid mater. Lymphatic vessels are closely associated with these nerve bundles and in mice they extend through the cribriform plate into the CNS-side of the skull, and potentially near these meningeal gaps. CSF and immune cells can move along this peri-nerve space into the periphery. **(C)** Rostral Migratory Stream (RMS). The RMS is an intra-parenchymal migration pathway for neural stem cells and evidence also suggests it facilitates the migration of dendritic cells. Starting at the subventricular zone, ISF and cells have been demonstrated to move toward the olfactory bulb along this route. Astrocytes and blood vessels populate this pathway, which are proposed to facilitate this fluid and cell movement. **(D)** Basal Dural Lymphatics. Here lymphatic vessels in the basal dura are proposed to facilitate immune cell egress and connect to a wider dural lymphatic system. Their close association to basal skull foramina provides a potential route to exit the skull, but similar to dorsal dural lymphatics, their communication with cells in SAS is still debated. **(E)** Choroid Plexus. The choroid plexus is a vascularized organ that produces CSF in the ventricles of the brain. Highly studied for its role in immune cell infiltration into the CNS, there is also limited evidence that it may participate in T cell egress from the CSF. Figure Acronyms: CSF, Cerebrospinal Fluid; ISF, Interstitial Fluid; SAS, Subarachnoid Space; OB, olfactory bulb; sCLN, Superficial cervical lymph node; dCLN, Deep cervical lymph node.

Generally, the lymphatic system has two primary functions within tissue it integrates; (1) to regulate fluid homeostasis and (2) to regulate immune function. Notably, the brain diverges from this model as it lacks a conventional lymphatic system within the brain tissue itself. Since lymphatic vessels only surround and do not penetrate the parenchymal space, there are several proposed routes immune cells infiltrating the brain can use to reach the peripheral lymphatic system, with many of these routes directly aligned with established fluid outflow pathways from the CNS. Here we will outline a few of the potential routes that immune cells have been demonstrated or are hypothesized to use to move within and out of the CNS. However, it is important to keep in mind that the passage of immune cells across the blood-brain barrier (BBB) in either direction is tightly restricted, and routes that drain fluid and proteins may not necessarily drain living immune cells at all barrier regions. Immune cells are significantly larger in size than fluid and solutes, and do not have the advantage of specialized cells in the CNS like astrocytes to facilitate them across barriers. Nevertheless, the interactions between the CNS exchange systems of the blood, cerebral spinal fluid (CSF), interstitial fluid (ISF), and meningeal lymph fluid compartments provide hints to the immune cells’ exit strategy and at the very least antigen access to immune cells in meningeal compartments. A visual summary of several of these fluid and immune cell pathways can be found in **Figure 1**.

### 2.1 Meningeal layers and arachnoid villi

**Figure 2:**
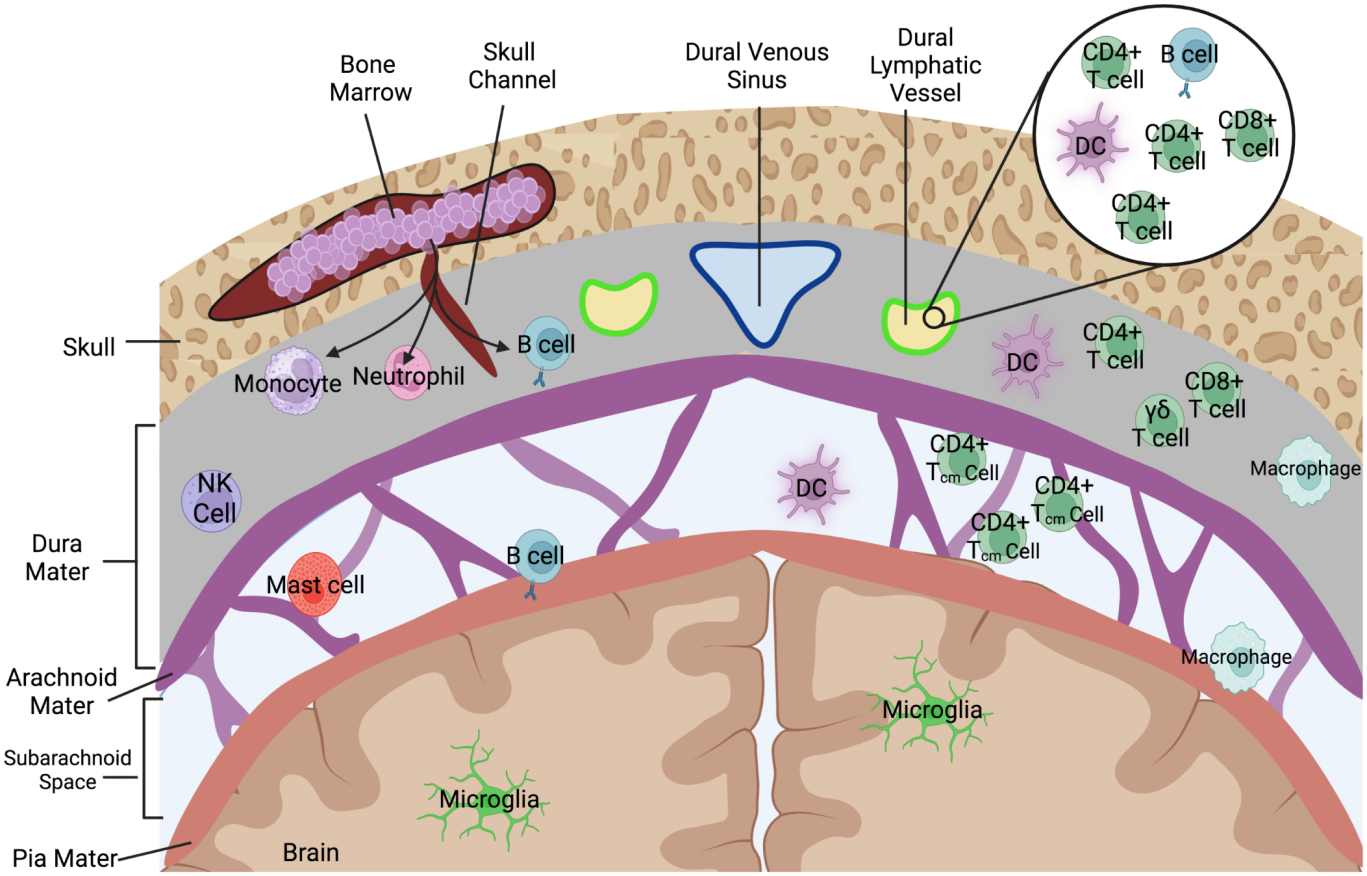
Immune cells that populate the meningeal spaces. A simplified schematic detailing the diverse repertoire of immune cells during homeostatic conditions at the dorsal meningeal spaces. The subarachnoid space is largely populated with T cells, specifically those with a CD4+ Tcm (Central memory) phenotype as well as some populations of myeloid cells including dendritic cells. Recent characterizations of skull bone marrow and dura have identified that immune cells can populate dural stroma without needing to first enter peripheral blood. Dorsal dural meningeal lymphatic vessels run alongside the superior sagittal sinus, and initial characterizations of the cellular composition of these meningeal lymphatic vessels have identified lymphocytes, DCs, and B-cells within the meningeal lymphatic lumen.

The meningeal layers are composed of three primary tissue components: (1) a fibrous dura mater directly beneath the skull, (2) the arachnoid mater, and (3) a thin pial layer that wraps directly around the brain parenchyma (**Figure 2**). Between the arachnoid mater and pia is the CSF filled subarachnoid space (SAS). An interesting anatomical phenomenon, particularly in humans, are arachnoid villi (or granulations) which are projections of this subarachnoid space via the upper arachnoid layer into the dura mater and into the dural stroma, including occasionally the dural venous sinuses (**Figure 1A**). While more prevalent in humans, arachnoid villi are proposed to ultimately allow for the flow of CSF into the dural sinus and at their discovery it was assumed they were the main route of drainage from the CNS. Over time their apparent role in CNS drainage has become less dominant for several reasons. How the flow of CSF occurs has been the subject of many hypotheses, but the strongest evidence presented belongs to a difference in pressure between the villi and dural sagittal sinus which creates the flow of CSF out of the CNS. Based on animal studies, some groups generally believe that the arachnoid villi route contributes little to overall drainage. For example, in Mollanji et al., the authors showed that when the cribriform is blocked in sheep, intracranial pressure builds up instead of dissipating by other routes (18). In rats, imaging studies implicate the spinal canal and olfactory routes not pathways involving arachnoid granulations (19). Surprisingly, some humans even appear to not have arachnoid granulations, and have otherwise normal CSF physiology (20), calling into question their necessity. Recent characterizations of arachnoid villi in humans, however, may have reestablished some of their immunological relevance. Shah et al., demonstrated that immune cells including DCs, B-cells, T cells, and macrophages populate the villi spaces, with select granulations appearing to penetrate into bone marrow spaces and adjacent to meningeal lymphatic structures with dura stroma (21). Indeed, the meningeal layers represent a rich immunological niche with several resident populations as well as infiltrating populations during inflammatory disease. A summary of the immune cells that populate the meningeal spaces can be observed in **Figure 2**. Nevertheless, more data is needed to fully understand each meningeal-associated immune cell population and their full contribution to CNS immune surveillance and inflammatory disease.

### 2.2 Dural lymphatic system

The study of the relationship between the lymphatic system and the central nervous system has been documented as early as the 18th century (22), but interest was reignited with the publications of several hallmark characterizations of the dural lymphatic vessels (23, 24). Meningeal lymphatic vessels have since been increasingly studied at several locations anatomically. Most studied are the lymphatic vessels which exist dorsal to the brain and adjacent to the superior sagittal sinus (**Figure 1A**), extending to the confluence of sinuses. At another dural site below the brain where the transverse sinuses extend laterally toward the base of the skull exists the basal dural lymphatics (**Figure 1D**). Together these vessels have been demonstrated to drain fluid, antigen, and cells to the draining cervical lymph nodes. As of this review, the meningeal lymphatic system appears to be one of the most promising routes of immune cell drainage from the CNS based on the available evidence, but with potential distinctions for each anatomical location and several gaps in knowledge. One gap is the exact mechanisms immune cells utilize to migrate from the CNS parenchyma into the subarachnoid space and then into meningeal lymphatic vessels. This is complicated further, because in some meningeal lymphatic locations such as the dorsal dural lymphatic vessels are by definition within the dura mater. As a result, immune cells within the subarachnoid space must feasibly transverse from across the arachnoid barrier layer and partial dura mater layers, which are relatively impermeable even to fluid (25). Nonetheless, characterizations of dorsal dural lymphatics through IHC do reveal a diverse immune cell population of CD3e+ cells (T lymphocytes), CD11c+ (DCs) and B220+ (B-cells) existing within LYVE+1 meningeal lymphatic vessels (**Figure 2**) (14, 24), but their origin is often debated. Channels, arachnoid villi, or regions where the dural lymphatic vessels may dip down into the sub arachnoid space have been proposed, and thus could feasibly facilitate this cell migration to dural lymphatics (21, 26). Additionally, during inflammatory states, it has been suggested that locally secreted proteases from activated immune cells in the meninges may assist in cell migration by weakening the extracellular matrix and tight junctions that presumably limit cell migration between meningeal layers under more steady state conditions. With that said, some groups still doubt whether a pathologically relevant connection exists between the dura and the underlying leptomeninges, particularly during autoimmune neuroinflammation (27).

The basal dural lymphatics are less understood but still characterized, initial imaging studies have shown anatomical connectivity between basal and dorsal lymphatic vessels along the transverse sinuses (28). One key feature of the basal lymphatic system is its close positioning to large skull foramen, which provide a gap to exit through the skull into the periphery, ultimately to the deep and superficial cervical lymph nodes (**Figure 1D**). Additional characterization of the basal lymphatics by Ahn et al., in mice argued that the thinner dural layer at this region, in combination with looser arachnoid membrane gives the basal lymphatic vessels more efficient drainage capabilities and access to SAS, which could translate to greater immune cell egress ability (28). It should be noted that directionality of flow from dorsal dural lymphatics to the basal dural lymphatics is not completely resolved in humans, as recent MRI flow mapping studies have indicated that the dorsal lymphatics along superior sagittal sinus flow from posterior to anterior, toward the front of the head (29, 30). This flow would be countercurrent to the dural venous sinus and some have argued that this could point to connectivity of the dorsal dural lymphatics to the cribriform route in humans, but more data is needed to confirm (29). Finally, an extensive network of lymphatic vessels can also be found associated with the vertebral column around the spinal cord. Recent characterizations have identified lymphatic vessels both in the epidural and dura mater space around the spine, which ultimately have connections to peripheral lymph node populations (31). However, while tracer studies have demonstrated the CSF draining capabilities of these spinal associated vessels, particularly in the lowest most sacral region (32), studies demonstrating the cell trafficking ability of these vessels from the CNS to peripheral lymph nodes are still lacking.

### 2.3 Cribriform plate lymphatic system

In addition to the dural lymphatics, there is also interest in meningeal-associated lymphatic vessels which exist near the cribriform plate near the front of the brain. The cribriform plate is located directly underneath the olfactory bulbs and is a perforated region of the skull between the brain and the nasal mucosa. These perforations allow the passing of olfactory nerves into the underlying nasal epithelium (**Figure 1B**). Of note, both pathogens and immune cells have been documented to travel along olfactory nerves, and it has long been hypothesized to be a major CSF outflow pathway from the CNS based on early animal studies (33–35). For example, Mollanji et al. performed surgery on sheep in which the cribriform plate was plugged using glue and found a near doubling of the intracranial pressure (18). Murtha et al. used CT imagining to come to a similar conclusion: CSF is drained mostly by the spinal canal and olfactory route, whereas other proposed pathways such as the arachnoid villi played only a minor role (19). The lymphatic vessels that are closely associated with olfactory nerves thus have been a focus of investigation for their role in not only fluid drainage but immune cell trafficking from the brain. Hsu et al. showed that during murine EAE there was an increase in cribriform lymphatic vessel area and number of dendritic cells bound to these lymphatic vessels (36, 37). It was also demonstrated that photoconverted cells from the brain drain and interface directly with cribriform plate lymphatic vessels (36). Anatomically, the cribriform lymphatic pathway is hypothesized to drain mostly to the superficial cervical lymph node (sCLN), whereas the dural lymphatic pathway may drain more directly to the deep cervical lymph node (dCLN) (14). In mice, these lymphatic vessels can be found on the CNS side of the cribriform plate (36). Importantly, recent characterization of olfactory nerves and meningeal layers in rodents have revealed discontinuity of arachnoid mater where lymphatic vessels interface with olfactory nerve fibers, potentially allowing for efficient fluid and immune cell egress at this location (**Figure 1B**) (37, 38). While more evidence is needed to fully understand the anatomy and role of these cribriform plate lymphatic vessels in humans, their unique relationship with cranial nerves and meningeal layers in mice may offer clues to how immune cells can exit the subarachnoid space at specific nerve-lymphatic hotspots.

### 2.4 Cranial nerves

The skull has several openings, or foramina, which mainly allow for the passage of blood vessels and cranial nerves out of the brain and into the periphery. With the skull itself being an obvious and significant barrier for immune cells leaving the CNS (even for cells already within the dural lymphatics), investigating immune cell drainage at anatomical locations with the largest gaps in the skull barrier only seems logical. As a result, there has been significant research into the interface between outwardly projecting cranial nerves, the meningeal layers surrounding them, and nerve adjacent lymphatic vessels could be the mechanism by which immune cells are reliably trafficked out of the CNS and toward cervical lymph nodes (**Figure 1B**). CSF has been shown to aggregate and drain out of the CNS along perineural sites of CSF outflow including optic nerves, olfactory nerves, and the nerves at the jugular foramina (glossopharyngeal, vagus, spinal accessory) (39–41). While the olfactory nerve pathway may be more dominant anatomically by proportional size in mice when compared to humans, other nerve pathways may have similar conserved functionality, such as with optic or trigeminal nerves (42). Additionally, in humans there is evidence that the meningeal lymphatic system and cranial nerves directly interface at key locations in the human skull foramina (42, 43). As a result, cranial nerves may offer a unique opportunity for immune cells to exit the CSF-filled subarachnoid space and interface directly with the peripherally connected lymphatic system.

### 2.5 Glymphatic system and perivascular space

The glymphatic system is a proposed mechanism that facilitates the movement of fluid and solutes into, across, and out of the CNS tissue space (**Figure 1A**). In essence, it is thought that the glymphatic system plays many of the roles of a traditional lymphatic system in the brain when it comes to parenchymal solute clearance. In 2012, Iliff et al. used two-photon microscopy and injected fluorescent tracers to elucidate the general route of the glymphatic system: CSF of the subarachnoid space flows into the brain parenchyma along para-arterial spaces, where this CSF along with ISF of the parenchyma can mix and flow out of the parenchyma along the paravenous spaces and where it could enter the bloodstream, stay in the subarachnoid space, or ultimately drain into the cervical lymph nodes (44). In these peri-arterial spaces, the CSF exists in a layer where it surrounds the vascular smooth muscle cells and is ensheathed by perivascular astrocytic endfeet. Iliff et al. also importantly demonstrated that AQP4, which is concentrated at these astrocytic endfeet, is essential for directing this CSF flux, as mice depleted of AQP4 showed greatly reduced clearance of injected tracers (44). How this influx of CSF is created is a byproduct of physiology. Iliff et al. and later Mestre et al. cited arterial pulsations as the driving force, van Veluw et al. pointed to vasomotion, Kiviniemi et al. reaffirmed arterial pulsations and added respiration, but importantly all of these factors contribute to directionality of flow (44–47). While the data on solute clearance ability of the glymphatic system is still building, evidence of glymphatic-dependent immune cell migration from the parenchyma to the CSF is less clear. Additionally, the glymphatic system as a whole is still controversial, with some showing support to (48) and others skepticism (49, 50). Importantly for this review, at the time of writing, most evidence is limited to tracking of tracers within the fluid, not cells (44). The glymphatic system could provide immune cells in the meningeal spaces access to solutes, antigen, and waste by transporting them from the parenchymal tissue to the subarachnoid spaces (48, 51). Immune cell aggregation and movement along the CSF-filled perivascular space, a component of the proposed glymphatic system, is more well understood and is characterized by populations of resident myeloid cells with the ability to stimulate infiltrating T cells (52, 53). Importantly, the perivascular fluid pathways may provide immune cells a needed connection from deeper brain tissue sites to the subarachnoid spaces above the cortical regions (**Figure 1A**).

## 3 Immune cells that migrate from CNS and meninges

While several immune cell populations are known to infiltrate into the CNS during disease states, only a few have sufficient evidence to outline the mechanisms of their potential migration from the CNS and the pathological consequences of this exit. The two immune cell populations with the most evidence of meningeal lymphatic draining capabilities are dendritic cells (DCs) and T cells, which will be extensively reviewed in the following sections. The ability of other immune cell populations like neutrophils and B-cells to migrate from the CNS will be assessed in less detail, as their evidence is less robust and, in some cases, controversial. Furthermore we will focus on peripherally derived immune cells; not resident immune cells like microglia, which are typically excluded in discussions of lymph node trafficking from the CNS, as injection experiments demonstrate their inability to migrate out of the brain (54, 55). A summary of key animal experiments which sought to test drainage capability of various immune cell populations from the CNS and meninges can be found in **Table 1**.

**Table 1:** Immune cell tracking experiments from CNS and meninges.

Immune Cell	Methodology	# Cells (Injection Volume)	Species	CLN	Olfactory/Cribriform Route	Dural Lymphatics	Other	Reference
**Dendritic Cells**	Intrathecal	2-5.0× 10^4^ (10 μl)	C57BL/6 mice	**Yes**	–	–	–	56
	I.C.	2.5× 10^5^ (30 μl)	C57BL/6 mice	**Yes**	–	–	–	57
	I.C.V.	3 × 10^5^ (10 μl)	Sprague Dawley Rats	**Yes**	–	–	–	58
	I.C.V	3 × 10^5^ (10 μl)	Dark Agouti Rats (EAE)	**Yes**	–	–	–	59
	I.C. (Parenchymal)	3 × 10^5^ (10 μl)	Dark Agouti Rats (EAE)	**No**	–	–	–	59
	I.C. Striatum	2.5 × 10^6^ (0.3 ul)	Lewis rats	**Yes**	–	–	**Blood**	55
	I.C.V.	1.0 x 10^6^	C57BL/6 mice (EAE)	**Yes**	**Yes**	–	**RMS**	8
	I.C.	2.5 × 10^5^ (20 μl)	C57BL/6 mice (EAE)	**Yes**	–	–	–	9
	I.C.M.	5 x 10^5^ (2 μl)	C57BL/6 mice	**Yes**	–	**Yes**	–	14
	I.C.V. (Ex vivo- fingolimod treatment)	1.0 x 10^6^	C57BL/6 mice (EAE)	***Reduced***	–	–	–	8
	I.C. (CCR7 -/-)	2.5 × 10^5^ (20 μl)	C57BL/6 mice (EAE)	**No**	–	–	–	9
	I.C.M. (CCR7 -/-)	5 x 10^5^ (2 μl)	C57BL/6 mice	**No**	–	**Yes**	–	14
**Monocytes**	I.C. entorhinal into lesion site	2.5 × 10^5^ (2 μl)	C57BL/6 mice	**Yes**	**Yes**	–	–	60
**Lymphocytes**	I.C.	2 × 10^7^ (100 μl)	Rabbit (German giant breed)	**Yes**	–	–	–	61
**T Cells**	I.C. entorhinal into lesion site	1 x 10^6^ (4 μl)	C57BL/6 mice	**Yes**	**Yes**	–	–	62
	I.C.V.	1 x 10^6^ (4 μl)	C57BL/6 mice	**Yes**	**Yes**	–	–	62
	I.C.M. (Naïve T cells)	1 x 10^6^ (2 μl)	Prox1-GFP C57BL/6 mice	**Yes**	**Yes**	**Yes**	–	14
	I.C.M. (CCR7 -/-)	1 x 10^6^ (2 μl)	Prox1-GFP C57BL/6 mice	***Sparse***	–	***Sparse***	–	14
	Intradural Photoconversion	N/A	C57BL/6 (donor KikGR BM)	**Yes**	**No**	–	–	14
	I.C.V. (Activated CD4 T)	2.5 × 10^5^ (2.5 μl)	C57BL/6 mice	–	–	–	**Choroid Plexus**	63
	I.C.M. (T_ova_)	1 x 10^6^ (25 μl)	Lewis Rats	***Sparse***	–	***Sparse***	–	27
	I.C.M. (T_MBP/_T_bSYN_)	1 x 10^6^ (25 μl)	Lewis Rats (EAE)	***Sparse***	–	***Sparse***	–	27
**B Cells**	I.C.M. CD19-Td tomato Splenocytes	2.5 × 10^6^ (5 μl)	C57BL/6 mice	**Yes**	–	**Yes**	–	64
**Microglia**	I.C. Striatum	2.5 × 10^6^ (0.3 μl)	Lewis Rats	**No**	–	–	–	55
**Neutrophils**	Two-Photon intravital microscopy	N/A	LysMGFP/+ mice	–	–	–	**Blood**	65

Studies include injections of immune cells via Intracisterna magna (I.C.M), intracerebroventricular (I.C.V.), or intracerebrally (I.C.) in animal models. "—", presence of immune cell in this location not analyzed or defined. N/A, not applicable.

### 3.1 Methodology of tracking immune cell migration from the CNS and meninges

Much of the evidence related to the tracking of immune cell migration out of the CNS relies on only a handful of methodologies. In general, the most widely used method involves the direct injection of fluorescently labeled immune cells either into the CSF via the cisterna magna (I.C.M) and intracerebroventricular (I.C.V.), or into the parenchyma through intracerebrally (I.C.) injection. The cells can then be tracked to specific anatomical locations within or outside of the CNS, such as the cervical lymph nodes. An obvious disadvantage of the above injection methods is the potential to directly disrupt the meningeal layers and introduce a non-physiologically relevant exit route at the injection site. Additionally, improper injection technique and excessive volume, can artificially elevate intracranial pressure and/or inflammation leading to deviations in homeostatic immune cell outflow pathways. Similarly, transgenic photoconvertible mice like KikGR (Kikume Green-Red) mice may offer the promise of non-invasive cell tracking in skin inflammation models through the usage of 405 nm light (66), but similar specific photoconversion in the CNS is still lacking. For one, parenchymal conversion of the immune cells often requires an injection site for a fiber optic cable to be inserted, and thus can be disruptive to the brain barriers. Secondly, even the more non-invasive transdural photoconversion methods which shine light through the skull non-specifically converts across several meningeal layers. Additionally, if bone marrow, arteries, or veins exist at photoconversion location through the skull, without proper controls, it will be exceedingly difficult to differentiate true immune cell migration from specific meningeal layers, such as between the leptomeningeal (pia and arachnoid mater) and dural layer. Current *in vivo* imaging techniques can be similarly disruptive, with many requiring cranial windows to surgically be created, and the depth of observation only limited to uppermost brain and meningeal regions (67). While there have been recent advances *in vivo* fluid and solute tracking assays, such as gold nanoparticle microtomography, which have allowed for better resolution in tracking of CSF outflow pathways from the CNS (68, 69), direct equivocation of these results to cell migration should still be cautioned. In summary, while the tools used to analyze immune cell migration from the CNS are improving, much of our current understanding is still built on imperfect techniques. Finally, transgenic KO mice and *in vivo* delivery of inhibitors have allowed for more careful investigations into the mechanistic pathways which dictate immune cell migration from the CNS toward the cervical lymph nodes. As we will see in the next sections, much of our understanding of the mechanisms that facilitate immune cell migration from the CNS are ultimately not novel biological processes, but instead have already been previously characterized in peripheral inflammation models. In contrast, the main difference between peripheral and CNS immune cell lymphatic migration, is how these pathways overlay upon the unique anatomical challenges presented by the brain, meninges, and skull. The next section summarizes the foundational evidence of CNS-emigrating immune cells from experiments utilizing these methods and the pathological implications.

### 3.2 Dendritic cells

DCs are the primary professional antigen presenting cells (APCs) of the body which can bind, activate, and regulate T cell and B cell populations. During peripheral tissue inflammation, activated CD11c+ migratory DCs which have phagocytized antigen, travel to draining lymph nodes to coordinate adaptive immune responses. Once in the draining lymph nodes, these migratory DCs can transfer antigen to lymph node resident dendritic cell populations (70), where it’s been estimated that a single lymph node DC can interact with up to 5,000 T cells every hour (71). While, most of these interactions are short lived adhesion interactions, such as the ones between LFA-1 on T cells and ICAM-1/ICAM-2 on DCs, when a DC presents cognate antigen to a naive T cell through major histocompatibility class type I (MHC I) or MHC type II (MHC II) complexes, the resulting activation cascade can produce large amounts of antigen specific T cells. These antigen specific T cells can adopt either a pro-inflammatory or tolerogenic role depending on the phenotype of the DC presenting antigen and the conditions within the lymph node niche (72). Thus, DCs represent powerful communicators of local inflammatory signaling with the broader peripheral immune system. As a result, in the context of neuroinflammatory disease, mapping the drainage pathway of antigen loaded DCs from the CNS and its related compartments has been an area of focus to better understand how the delivery of CNS-antigen to the cervical lymph nodes can influence diseases like multiple sclerosis and brain cancer (36). While soluble antigen from the CNS can reach draining lymph nodes via the meningeal lymphatics, less is known about cell-mediated antigen migration out of the CNS and whether this takes pathological precedence over non-cell mediated antigen delivery via drained lymph fluid. In this section we will review the literature regarding DC efflux from the brain, which remains to be the most well studied CNS-emigrating immune cell population with antigen delivery capabilities.

#### 3.2.1 Evidence of DC migration from the CNS and meninges

Steady-state populations of peripherally derived DCs exist within the meninges and choroid plexus, but the presence of infiltrating dendritic cells has scarcely been documented in the healthy brain parenchyma (73). Evidence suggests that most of the DCs that populate the steady state meninges and choroid plexus arise from circulating pre-DCs originally from the bone marrow which become conventional dendritic cells (cDCs) residing at these sites (73). During CNS-autoimmune disease, it is these cDCs at brain barrier sites (and not the bordering macrophages) which have demonstrated the ability to license T-cells into the brain parenchymal spaces (74). Additionally during neuroinflammation, DCs have been consistently documented to increase in the CSF of human patients (75–77) as well as the CNS parenchyma at sites of inflammation or damage (78). During neuroinflammation, many of the DCs in the CNS still display characteristics of cDCs, but non-conventional “DC-like” cells arising from monocytes (or moDCs) are also present and can have overlapping morphology and expression patterns (79–81). Evidence suggests moDCs have distinct trafficking ability and function in the lymph node which is still being characterized (see “Monocytes and Peripherally Derived Macrophages’’ section). As a result, experiments which more closely identify the origin, cell-subset, and cell-state of DCs which populate the CNS and meningeal spaces will be essential to dissociate how various DC populations coordinate CNS-related immunity to the periphery.

In line with this, recent investigations into peripheral inflammation models have revealed that while pre-DCs and their cDC progeny possess the ability to proliferate in their resident tissue environment, even clustering together under steady-state conditions, during inflammatory events much of the local increase in cDCs in the inflamed tissue arises from “infiltrating” pre-DC which have recently egressed from the bone marrow (82). Thus, inflammatory seeding of pre-DC into tissue, not proliferation from pre-existing local pre-DC and cDCs appears to be the main driver of cDC increase (82). In the context of the brain, this phenomenon of pre-DC seeding is still being investigated, but skull channels which connect bone marrow reservoirs to meningeal layers have been proposed as the main pathway for most of the seeding of myeloid cells in the meninges during both steady state and neuroinflammatory disease (83). Additionally, an important unresolved feature of the meningeal niche is how resident cDCs and recruited pre-DCs might engage in the meningeal microenvironment to further their own maturation/development, presumably through Flt3L related signaling interactions (73). Recently published data from the lymph node suggests that highly coordinated spatiotemporal networks of cDC1s in LN medulla regulates pre-DC maturation through the bioavailability of Flt3L during inflammation (84), but to what extent similar anatomically defined networks exist in other tissues such as the immune rich meningeal layers is still unclear.

Nonetheless our lab showed that peripheral DCs can migrate into the CNS across barrier sites via a variety of chemokine gradients, namely the CCR2-CCL2 signaling pathway (85). Once in the CNS, DCs can perform a variety of functions within the local CNS tissue environment such as restimulate co-infiltrating T cells or dampen local immune responses (7). Of these infiltrating DCs, it is generally accepted that a subpopulation of DCs can migrate out of the brain. In line with this is evidence from our lab that when antigen loaded GFP^+^ DCs are injected into the brain parenchyma of mice, a subpopulation of these injected DCs can be detected in cervical lymph nodes 7 days later (57). It should be noted that only a fraction of the injected DCs were measured within the cervical lymph node at this time point (approximately 0.5-1%), but nonetheless this was enough to initiate antigen specific T-cell generation within the lymph node. Hatterer et al., further demonstrated in healthy mice that after intra-CSF injection of fluorescently labeled dendritic cells, DCs could be observed in the cervical and axillary lymph nodes 3 days after injection (58). Here they found that intraparenchymal injections of DCs resulted in little to no measurable migration out of CNS, indicating that DC drainage could largely depend on CSF outflow pathways. The same group demonstrated that during EAE, CSF injected DCs migrate to cervical lymph nodes at a higher rate, as well as infiltrate into the brain tissue at lesion sites (59). As a result, it appears that DC populations within the CSF contain both parenchymal infiltrating DCs as well as migratory draining DCs in transit to lymph nodes, and that neuroinflammatory state plays a role in drainage capacity of the CNS.

Louveau et al., demonstrated that significant amounts of CD11c+ DCs can be observed within the dorsal dural lymphatic vessels (**Figure 1A**) (24). Additionally, the same group later reported that after injection of stimulated DCs into the CSF via the cisterna magna, the labeled DCs aggregated within the lymphatic system near the transverse sinus and later in the dCLN (14), demonstrating the role of these meningeal lymphatic vessels in facilitating DCs from the CSF. It has been indicated by Rustenhoven et al. that migratory DCs populate the dural stroma near the dural sinus, and can directly access antigen from the CSF through previously proposed solute pathways (**Figure 1A**). This would potentially allow for antigen sampling by DCs in the outermost meningeal layer without directly having to infiltrate into deeper tissue layers (86). Furthermore, recently characterized skull to dura channels have been proposed to allow for these dural regions to be homeostatically populated by myeloid cells in the nearby skull bone marrow, including dendritic cells, and thus allow for DCs to be in the meningeal spaces without direct recruitment from the blood (**Figure 2**) (83). These non–blood-derived myeloid cells were reported to also be recruited to the dural spaces during neuroinflammation, and potentially have a more regulatory role than their blood-derived myeloid counterparts (83). It should be noted however that recent studies have asserted that the underlying leptomeningeal (pia and arachnoid) spaces still have greater antigen availability and APC stimulatory ability when compared to dural populations, particularly during autoimmune neuroinflammation (27, 87). Together this evidence suggests that the antigen presenting cells along leptomeningeal vessels likely dictate much of the brain tissue infiltrating ability of pathogenic T-cells which have undergone transendothelial migration at this site (74, 87). Merlini et al., even argues that the dura is essentially “excluded” from the autoimmune inflammatory process due to their lack of antigen access (27), but others assert homeostatic dural CSF and antigen sampling plays a role under more steady state conditions (51).

Interestingly, within the actual brain tissue itself, data suggests CD11c+ cells aggregate within the inflamed CNS tissue in a pattern across the parenchymal space (at least in mice). For example, during CNS autoimmune disease, CD11c+ cells within the brain have been documented to accumulate near ventricular spaces and are hypothesized to travel along the rostral migratory stream (RMS) in the murine EAE model (**Figure 1C**) (8, 88). The RMS is an intraparenchymal migratory pathway primarily known for its role in movement of neuroblasts from their origin (subventricular zone) to their termination point at the olfactory bulb. The RMS is characterized by both the abundance of astrocytes and blood vessels, which are hypothesized to assist in the unique migratory properties of this pathway (89, 90). As a neuroblast highway, the RMS is most active during development, however, evidence in both adult mice and humans suggests that the olfactory system remains one of the few sites (along with the dentate gyrus) in the adult brain that could facilitate neurogenesis (91, 92). It should be noted, however, that while well established in mice, adult neurogenesis in humans remains to be a controversial field with several conflicting studies (93, 94) and the role of the RMS in human immune cell migration is still not fully understood. Nonetheless, immunohistochemistry analysis from our lab and others have reported that in during EAE, CD11c+ accumulate in large numbers at the olfactory bulb, and can be located near olfactory nerves that extend out of the brain (**Figures 1B, C**) (8, 36, 85, 95, 96). However, what percentage of these CD11c+ cells along RMS and olfactory bulb regions are true intraparenchymal migrating DCs remains to be fully demonstrated, as CD11c cells in the brain represent a heterogenous group and can also be expressed on a variety of immune cell populations including microglia (74, 80).

These nerve projections that pass through the olfactory foramina of the cribriform plate have long been hypothesized to be a major outflow pathway for both fluid and immune cells into the periphery (15, 97). Furthermore, recent murine studies by our lab and others have indicated that the E-Cadherin positive arachnoid mater becomes discontinuous alongside these olfactory nerves, precisely when they interface with CNS side cribriform lymphatic vessels (37, 38). As a result, the olfactory nerve/lymphatic interface could provide a uniquely positioned outflow pathway where cells traveling in perineural space near the subarachnoid CSF can exit near layers along nerve fibers and interact with lymphatic endothelial cells (LECs) at the cribriform plate (**Figure 1B**). In agreement with this, when the phenotype of immune cells interacting with cribriform lymphatic vessels was analyzed during murine EAE, CD11c+CD11b+ dendritic cells dominated the lymphatic interacting populations (37). Finally, evidence suggests that other cranial nerves may serve as exit pathways for DCs, for instance our lab has observed large accumulations of CD11c+ immune cells along optic nerve pathways leading out of the brain (96).

#### 3.2.2 Receptors of DC migration out of the CNS and meninges

Similar to DCs in peripheral tissues, it’s thought that CCR7 is highly involved in DC migration from the CNS toward the draining cervical lymph nodes. CCR7 is a potent receptor of DC migration toward the ligands CCL19 and CCL21, which are highly expressed by lymphatic vessels. DCs typically upregulate CCR7 after the uptake of antigen and/or upon toll-like receptor activation. This CCR7 upregulation in DCs is instrumental for DC trafficking to lymph nodes, but it represents just one component of a much wider expressional cell state that migratory DCs adopt as they uptake antigen and move away from proinflammatory signals toward lymph nodes (98, 99). Kivisäkk et al. first reported the presence of CCR7+ DCs in the brain and CSF of human MS patients, finding that a significant amount of CCR7+ DCs aggregated at lesion sites (76). The same researchers found that the CCR7 ligands CCL19 and CCL21 were absent at these lesion sites, but it should be noted however that there have been several reports of CCL21/CCL19 expression at lesion sites (100, 101), which indicates that CCR7 ligand signaling within the brain exists and could be involved in the infiltration process. There is also evidence that CCL19 is increased in the CSF of patients with neuroinflammatory diseases, which is hypothesized to be relevant to the process of migration within the subarachnoid space (102).

CCR7+ DCs have since been reported in meningeal lymphatic drainage sites at the cribriform plate lymphatics (**Figure 1B**) (36). Importantly, investigations into the lymphatic vessels within the dura and at the cribriform plate have revealed that brain adjacent lymphatic vessels express CCL21+, which feasibly provides a gradient to facilitate DC migration at these sites toward peripheral lymph nodes (14, 23, 36, 86). ScRNAseq data suggests that fibroblast-like cells within the dura also express CCL19, providing additional CCR7 mediated signaling within meninges, but it is still unclear whether this is related to egress out of the CNS (86). Even so, while the gradients of CCR7 ligands within the CNS have not been fully mapped out, it appears CCR7 signaling is still one of the primary mechanisms that allows for a subpopulation of DCs to ultimately leave the brain compartments. Clarkson et al. highlighted the importance of CCR7 on DCs during EAE, where it was demonstrated that stimulated intracerebrally injected DCs lacking CCR7 (but pre-loaded with myelin antigen), failed to migrate from the brain to the cervical lymph nodes. Whereas in a parallel experiment, intracerebrally injected CCR7 WT DCs could be found at the draining cervical lymph nodes 7 days after injection (9). These results were recapitulated by Louveau and Herz et al., when CCR7-deficient DCs failed to drain to dCLNs from their injection site in the CSF (14).

Another DC receptor of note is CXCR4, which according to a study by Mohammad et al. could facilitate the intraparenchymal migration of DCs across the RMS toward a potential DC draining site at the olfactory-cribriform plate axis (8). CXCR4, which coincidentally also allows neural progenitor cells to be guided toward the olfactory bulbs via a CXCL12 gradient in the brain (103, 104), may also guide migratory DCs along the same intraparenchymal pathway (**Figure 1C**). In the periphery, CXCR4 has also been shown to facilitate trafficking of skin DCs toward afferent lymphatics (105). While it’s thought CCR7 is a more potent receptor of migration, CXCR4 like CCR7, becomes upregulated in mature DCs (106), making it a worthy receptor for investigation of migratory DCs in the brain. In agreement with this, anti-CXCR4 treatment with AMD3100 in the CNS leads to accumulation and retention of CD11c+ cells in the brain (8), but again further characterization of these CD11c+ cells is likely necessary to identify them as true migratory DCs. Cellular sources of CXCL12 along the RMS could be from a variety of resident cells within brain, as several cells types within the brain have been shown to express CXCL12, including microglia and neurons, but astrocytes are heavily implicated in establishing the gradient due to their abundance at the RMS and demonstrated ability to upregulate CXCL12 during neuroinflammation (107). For example, in a viral model of multiple sclerosis, GFAP+ astrocytes were shown to be the primary producers of CXCL12 during chronic inflammation in the CNS (108). It remains to be seen if other chemoreceptors involved in neuroblast RMS migration have overlapping functions with migratory DCs along this pathway. For example, at the termination of the RMS in the olfactory bulb, the detachment of migrating neuroblasts from the RMS has been shown to be regulated by Reelin secreted by mitral cells that populate the periphery of the olfactory bulb (109). Engagement of Reelin receptors like ApoER2 then allows neuroblasts to transition toward a more radial migration pattern toward outer layers of the olfactory bulb (**Figure 1B**) (110). In our lab we have observed that during EAE CCR7+ DCs in the olfactory bulb also express ApoER2, but it is unclear whether DCs in the olfactory bulb have co-opted yet another neuroblast migration signal to facilitate their exit from the brain. Recent evidence suggests ApoER2 on myeloid cells is indeed relevant to EAE progression (111). Alternatively, non-parenchymal locations also have measurable levels of CXCL12. For instance, in MS patients, the CSF has been shown to have detectable levels (112). Additionally, scRNAseq data has also reaffirmed that stromal cells in the meningeal spaces express CXCL12 (86, 113), which could also mediate DC migration toward outermost meningeal layers.

Additionally, Mohammed et al. heavily implicates sphingosine-1-phosphate receptors (S1PR) in the egress of DCs from the brain (8). The ligand for S1PR, the lipid S1P, is a potent mediator of T cell exit from lymph nodes, with systemic delivery SP1R blocking drugs like Fingolimod preventing MS pathology through increased retention of lymph nodal T cells (114). Mature DCs can use S1PR to facilitate migration to lymph nodes (115–117) and importantly Fingolimod delivered to ventricles in EAE mice lead to aggregation of CD11c+ cells in the olfactory bulbs near the cribriform plate, and ultimately impaired overall drainage to CLN (**Figure 1B**) (8). Similarly, when experimenters treated DCs with fingolimod and subsequently injected into the brain, it resulted in a reduction in DCs ability to reach CLNs. Together, the resulting evidence suggests DCs in the brain utilize many of the same receptors observed in peripheral migration schemes, but significant evidence is needed to fully map out the complete mechanism of CNS-egress, particularly when it comes to DCs at different anatomical locations in the brain.

#### 3.2.3 Pathological consequences of modifying DC emigration from CNS and meninges

Failure to properly drain DCs from the brain could have a variety of pathological implications during neuroinflammatory disease. One such complication is that retained DCs could accumulate within the brain and exacerbate CNS inflammation. Since DCs within the CNS can present antigen and restimulate co-infiltrating T cells, retained DCs could lead to pathogenic T cell response depending on their phenotype. This phenomenon was documented by Clarkson et al., in which EAE clinical scores were significantly higher in mice that were injected with CCR7-KO DCs when compared to WT DCs. Here, levels of pathogenic IFNγ and IL-17 producing CD4+ T cells were substantially increased in the brain, giving evidence that sustained pro-inflammatory stimulation of infiltrating immune cells was occurring via retained DCs (9). Mohammad et al., found similar results when fingolimod, a mononuclear cell-sequestering drug, was delivered directly to the RMS (**Figure 1C**). Here they found an increase in retained CNS DCs during EAE, which resulted in worse EAE clinical scores. However the authors gave evidence that this pathogenic effect was not entirely the result of elevated local T-cell restimulation in the CNS, but rather the result of inhibiting the migration of RMS migrating tolerogenic DCs in the brain that would otherwise have drained and elevated Treg activity in the lymph node (8). In agreement with this theory, they found that cervical lymph node Treg activity was decreased in RMS-fingolimod treated mice (8).

Thus, one primary focus of migratory DC drainage from the brain is their influence on downstream immunity in the cervical lymph node. Here data suggests that delivery of CNS-antigen by migratory DCs to lymph node resident DCs can drive immunogenic and tolerogenic proliferation of lymphocytes, which can then shift the peripheral pool of adaptive immune cells which ultimately populate the blood and traffic to the CNS. In agreement with this, myelin-containing APCs can be found in the CLN of marmoset and rhesus monkeys with EAE as well as patients with MS (118) and excision of dCLNs can partially reduce severity in murine EAE (12, 119). Furthermore, our lab showed that I.C. injection of OVA-loaded DCs drain to the CLN and prime OVA-specific T-cells (57). Additionally, as mentioned in Mohammad et al., the tolerogenic impacts of APC mediated CNS-antigen delivery to cervical lymph nodes may also play a role in suppressing immune responses (8) and interestingly the superficial cervical lymph nodes have established roles in mediating tolerance of nasally administered antigen (120). So, while the cervical lymph nodes may be the anatomical end point of CNS draining DCs, the resulting interactions between the delivered antigen and lymphocytes in the CLN can have lasting effects on both peripheral and CNS immunity.

As a result, improper DC migration from the CNS may lead to a variety of deficits in immune surveillance of the brain and potentially neuropathology. For example, in the context of brain cancer, the delivery of tumor antigen from the CNS to the periphery is known to play an important role in the control of brain cancers via the creation of anti-tumor T cells in the draining lymph nodes (121). Interestingly, a recent report suggests that soluble tumor antigen drainage from the CNS alone may not be enough to facilitate an efficient antitumor T-cell response in a mouse model of glioma, and that the cell mediated delivery of antigen by CNS-draining DCs is essential to anti-tumor immunity (122). Illustrating this, Hu et al. found that while VEGF-C induced meningeal lymphangiogenesis allows for larger anti-tumor T cell response against brain tumors and thus increased survival, when mice were given a CCR7 or a CCL21 blockade in combination with VEGF-C, the resulting disruption in DC migration abolished any survival benefit from the VEGF-C (pro-lymphangiogenesis) therapy (122). Together, this study indicates that the anti-tumor survival benefit observed from VEGF-C therapy could be the result of the increase in antigen-loaded DC migration from the CNS, not simply increased soluble antigen drainage from the bulk expansion of meningeal lymphatic vessels. It should be noted that the overall contribution of APC mediated versus soluble antigen delivery from the CNS and meninges still remains an open question, but evidence from the periphery suggests that the route of antigen delivery can dictate differential aspects of immunity (123–126). Nonetheless VEGF-C appears to increase antigen delivery via both routes (APC mediated vs soluble). In agreement with this, VEGF-C has also been shown to increase CCL21 expression in expanded lymphatic vessels, thus providing a mechanism to directly enhance DC migration pathways from the CNS and antigen stimulation in the lymph nodes (122, 127). Interestingly, our lab found that DCs (along with other CD11b+ macrophage populations) at the cribriform plate (**Figure 1B**) produce VEGF-C in close proximity to LYVE-1+ cribriform lymphatic vessels during neuroinflammation, directly stimulating lymphangiogenesis and likely assisting in both cell-mediated and soluble antigen delivery to lymph node (36).

Conversely, inhibiting VEGFR3 signaling in meningeal lymphatic vessels may offer a way to therapeutically restrict CNS-DC outflow to cervical lymph nodes, through the dampening of both lymphangiogenesis and migration related signaling cascades. Use of VEGFR3 inhibitors has been shown to reduce EAE clinical scores (36), presumably through decreased CNS antigen delivery which is required for antigen specific CD4+ T-cell proliferation in the cervical lymph nodes. Similarly, visudyne ablation of meningeal lymphatic vessels also had a similar effect, with a delay in EAE development which coincided with reductions of DCs interacting with myelin specific T cells in the deep cervical lymph nodes (14). However, anatomical specificity and more specific targeting of meningeal lymphatic function may be needed to fully understand each region’s contribution to disease progression. Of note is a recent publication by Li et al., in which experiments using VEGFR3-blocking antibody, LEC-specific *Vegfr3* deletion mouse model, and VEGF-C/D trap, to inhibit dural meningeal lymphatic function have downplayed the contribution of dural lymphatics to pathogenic antigen drainage from the CNS to cervical lymph nodes during autoimmunity (108). Instead the authors argue that increased focus should be placed upon the leptomeningeal immune niche, with its greater antigen availability and more stimulatory APCs. One interesting finding from Li et al., was that the dural lymphatics experienced no expressional nor morphological changes during EAE (128). Lack of dural lymphangiogenesis during EAE has been previously reported by multiple labs (14, 36), and could point again to their distance from inflammatory signaling, particularly VEGF-C secretion by immune cells or an overall differential response to VEGF-C/VEGFR-3 signaling. Interestingly lymphangiogenesis at the cribriform plate during EAE is consistently observed (14, 36, 37), but it is unknown how the lymphatic inhibition methodology used in Li et al., impacted antigen and DC drainage specifically along the olfactory route or other less studied routes. Interestingly, the LEC specific *Vegfr3* deletion experiments did result in accumulation of immune cells in the dura, and a reduction in drained antigen and T-cell activation in the cervical lymph nodes, implicating participation of dural lymphatics in cell and antigen drainage, however these reductions did not dampen EAE scores (128). As a result, one interpretation of these results is that there is likely a compensatory balance between lymphatic independent pathways within the CNS and lymphatic dependent pathways which ultimately dictates pathogenic CNS immune response. Indeed, the cervical lymph nodes are just a single component that could influence the potential immune response in the CNS. Illustrating this recently, Kovacs et al. reports in an toxoplasma gondii brain infection model, that while ligation of both deep and superficial lymph nodes reduced co-stimulatory DC phenotype and antigen specific T-cell generation in the CNS-draining cervical lymph nodes, the brain was still able to coordinate a sufficient immune response against the pathogen (129).

#### 3.2.4 Modifying phenotype of migrating DCs at meningeal lymphatics

One issue with the therapeutic manipulation of APC drainage or migration from the CNS, as seen in the experiments in the previous section, is that while draining CNS DCs may have overlapping mechanisms of migration (CCR7, exit pathways, etc), these migrating DCs may not all share the same stimulatory phenotype once in the lymph node. In essence, migrated DCs can act in either a tolerogenic or autogenic context depending on their expressional makeup. Fate determination of activated T cells in the lymph node is coordinated by APCs via the molecules they secrete during T cell priming interactions, and alterations in these secreted factors can produce widely different effector T cell subtypes. For example, IFN-γ produced by DCs during priming of naive T cells can shift T cells toward Th1 phenotype, whereas TGF-B and IL-2 secretion by DCs can generate anti-inflammatory Treg subsets (72, 130). Additionally, recent data from peripheral models has shown that antigen transfer from migratory DCs to LN resident DCs is “co-encoded” with contextual cues from the upstream tissue environment (131). These cues from the migratory DCs come in the form of transferred PAMPs which act upon TLRs on the surface of LN resident DCs, coordinating tissue specific T-cell responses (131). As a result, any functional heterogeneity among migrating DC populations can have wide impacts, and simply adjusting the biological dial to increase or decrease bulk flow of APCs from the CNS and meninges may not have the intended therapeutic effect. In multiple sclerosis for instance, restricting meningeal lymphangiogenesis and DC outflow via VEGFR3 inhibitor may potentially prevent the creation of autoreactive myelin specific T cells, it could also stifle the drainage of subpopulations of tolerogenic DCs. In cancer models for example, it has been shown that some tumor microenvironments can alter DC expressional profile to a more tolerogenic phenotype, leading to reductions in effector T-cell stimulatory ability and/or greater Treg proliferation in the lymph nodes, thus stifling anti-tumor immunity (98, 132).

So, while heterogeneity of DC phenotypes may complicate therapeutic benefit of broadly targeting immune cell drainage, it also introduces the potential to modify or target APCs at drainage “choke points” to skew APCs (and thus effector T cells in LN) to the most therapeutically beneficial phenotype. Our lab is particularly interested in the upstream modification of migratory DCs at the meningeal lymphatics by the lymphatic endothelial cells themselves. A recent finding by our lab discovered that the afferent lymphatic vessels at the cribriform plate (**Figure 1B**) upregulate molecules related to leukocyte cross-talk during EAE, with neuroinflammation appearing to upregulate cell to cell adhesion molecules like VCAM-1, as well as tolerogenic molecules like PD-L1 within the lymphatic cells themselves. DCs appear to get retained and aggregate at the cribriform lymphatics potentially allowing for long-term signaling interactions to take place. Moreover, when these PD-L1+ LECs were cocultured with myelin specific T cells, they induced greater cell death, demonstrating functional signaling ability (37). In summary, the role of meningeal lymphatic vessels continues to grow across a number of diseases, and recent data suggests that treating these drainage sites as more than simplistic “tubes” that facilitate clearance, and rather as sites of active immune regulation may ultimately provide a better framework to develop new therapeutic strategies.

### 3.3 T cells

Steady state T cells in the brain parenchyma are scarce, and generally undetectable. In the meningeal spaces, however, their presence is more permissive, with naive T cell populations circulating. During neuroinflammatory states T cells can infiltrate across the brain’s barrier in considerable numbers. This process is thought to be mediated by co-stimulation of local APCs at the brain’s borders, and is extensively explored in the following reviews (133, 134). During diseases like multiple sclerosis, it’s thought that most T cells that infiltrate the CNS during neuroinflammation likely do not migrate out. As in peripheral tissue, there exist coordinated pathways of cell apoptosis to control T cell populations locally in the brain and contain inflammation. For example, FasL ligands or PD-1/PDL1 stimulation can all easily trigger cell death within the brain compartments to limit T cell activity locally. Microglia and co-infiltrating DCs express inhibitory PD-L1 as well as Fas receptors to regulate T cells within the brain. However, in certain contexts when large amounts of T cells are infiltrating into the CNS, subpopulations of T cells may migrate out as seen in peripheral inflammation.

#### 3.3.1 Evidence of T-cell migration from CNS and meninges

Goldmann et al., 2006. first demonstrated that when GFP+ T cells (5x10^6^ cells) were injected into brain lesion sites, fluorescent T cells could be observed at the deep cervical lymph nodes within 12 hours of injection, with large populations appearing at 24 and 48 hours post injection (62). In line with outflow pathways at the nasal lymphatics, at 12 to 24 hours after injection these GFP+ T cells aggregate along olfactory nerves at the cribriform plate (**Figure 1B**). This observed drainage of T cells occurred regardless of lesion status, with non-lesioned animals exhibiting similar pathways of drainage, and regardless of activation status of the injected T cell (62). It should be noted, however, that many of T cells that infiltrate the brain during disease are typically activated and antigen specific. During EAE, early *in-vivo* imaging studies reported that some of the infiltrating effector CNS T cells can exhibit “stationary’’ kinetic behavior once within the CNS which appears to be directly related to their antigen restimulation by local APCs (135). Many of the T cells however (~upwards of 2/3) moved quickly and somewhat randomly throughout the tissue, with non-pathogenic T cells appearing to move most freely within the CNS (135). Later experiments by our lab utilizing 2D2 myelin specific T cell mice across multiple day time periods observed T cells aggregating in close proximity DCs along the rostral migratory stream (**Figure 1C**). Here, the highest colocalization of T cells and DCs occurred in the olfactory bulb, where approximately 55% overlapped (88).

The meningeal spaces have long been understood to have a substantial population of T cells (**Figure 2**), particularly during neuroinflammatory states and while T cells have recently been reported inside dorsal dural (24), basal dural (28), and cribriform lymphatics (37), their migratory relationship through the meningeal lymphatics is still being mapped out. In 2018, Louveau and Herz et al. offered an extensive characterization of T cell emigration from the CNS through the dorsal dural lymphatic system (**Figure 1A**) (14). They achieved this in part through the utilization of KikGR photoconvertible mice, and illuminating the meningeal spaces through the intact skull. Here the authors observed converted T cells accumulating primarily in the dCLNs of the converted mice. Furthermore, the same group injected fluorescently labeled T cells I.C.M. into the cerebrospinal fluid. Flow cytometry analysis revealed that similar absolute numbers of injected T cells reached the dCLNs and sCLNs, but the percentage of GFP+ T cells was higher in the dCLNs compared to the sCLNs, peaking around 12 hours post injection. When the dorsal dural lymphatic vessels were ablated however (**Figure 1A**), the ability of injected T cells to reach the dCLN was significantly inhibited. Emigrating T-cell could also be observed along the nasal route (**Figure 1B**), but the group argues that it represents only a minor route for I.C.M. injected T cells (14). Recently however the ability of T cells to migrate from the CSF to the dural lymphatics have been called into question, as the I.C.M. injection of both CNS antigen specific and non-specific T cells revealed only occasional egress into dura and dCLN, mostly aggregating in the leptomeningeal spaces (27).

Along with cranial nerves and the meningeal lymphatic vessels, another site of T-cell exit from the CSF may occur at the choroid plexus. The choroid plexus is an organ that exists within the CSF filled ventricles of the brain and serves as a boundary between the blood and the CSF (**Figure 1E**). Largely studied for its role in CSF production and fluid regulation of the CNS (136), the choroid plexus is also known to be an important site of immune cell entry into the CNS from the peripheral blood (137). For example, during EAE, T cells within the fenestrated capillaries of the ChP have been shown to infiltrate into the CSF by crossing through the outer epithelial barrier, which directly facilitates disease progression (138). Much of the immune cell trafficking at the ChP is thought to be dominated by lymphocytes, which is supported by evidence that the CSF is largely dominated by T cells (139, 140). Recent publications indicate the choroid plexus may actually function as a bidirectional pathway for immune cells, in which immune cells can enter from the CSF into the ChP stroma. This was demonstrated by Strominger and colleagues (63), where intracerebroventricularly injected activated CD4 T cells were found to home to, adhere to, and enter the ChP from the CSF (**Figure 1E**). Epithelial expression of leukocyte adhesion molecules like ICAM-1 on ChP epithelial cells was thought to facilitate this T-cell entry (141, 142). Once within the ChP the authors report that the T cells were stimulated by local ChP APCs and proliferated, highlighting this region as a potential immunologically active drainage site. While still controversial, whether or not other immune cells within the CSF, like DCs can also reenter the ChP is still unknown and will require additional studies to verify the pathological contribution of this migration during disease.

#### 3.3.2 Receptors of T-Cell Migration from CNS and meninges

Like DCs, it has been well established that subpopulations of T cells at peripheral sites of inflammation can also express high levels of CCR7 and migrate via afferent lymphatics (143, 144). In the CNS, characterization of human CSF has revealed that CCR7 expressing T cells populate the healthy subarachnoid space (145). It is thought that most of these are central memory T cells which patrol the brain’s borders as a mechanism of immune surveillance (**Figure 2**) (145, 146). Louveau and Herz et al., first investigated the role of CCR7 in meningeal lymphatic drainage of T cells, where they reported up to 40% of the steady-state meningeal CD4 T cells expressed CCR7 (14). Here the authors injected 1:1 ratio of CCR7 KO and CCR7-WT T cells into the cisterna magna and found that the migratory ability of CCR7-KO T cells to both the dural lymphatics and deep cervical lymph nodes was significantly inhibited after loss of CCR7 (14).

Additionally, as mentioned in the previous section, some T cells in the CSF may possess the ability to reverse migrate into the ChP (**Figure 1E**) (63). There is also some evidence that the ChP expresses CCL19 (100, 147) which could imply CCR7 mediated chemotaxis, but this is still controversial. Strominger et al. illustrates that during LPS induced neuroinflammation, ICAM-1 is upregulated on ChP epithelial cells and T cell trafficking into the ChP organ from the CSF is in part ICAM-1 dependent (63). As for other T cell receptors, CXCR4, a key receptor of DC migration across the RMS, is also reported to be highly expressed within the myelin specific CD4 T cell populations during EAE (148). CXCR4 receptor has recently been demonstrated to mediate periphery T cells toward lymphatic structures in cancer models (149) and to the bone marrow in EAE (148).

#### 3.3.3 Pathological consequences of modifying T cell emigration from CNS and meninges

Just as with DCs, improper clearance or disrupted migration of T cells from the CNS compartments could have pathological implications. One theory is that migration of T cells to the periphery could play an important role in the local immune homeostasis of the meninges. Demonstrating this, a recent publication by Da Mesquita et al. reports that CCR7 expression by T cells is significantly reduced in aging mice, which coincides with elevations of Treg activity in the meninges (**Figure 1A**) (10). Here the authors argue that inability to effectively drain CCR7+ Tregs from the meningeal microenvironment results in a pathological increase in Treg response in the CNS, which has been shown by others to negatively affect amyloid beta (Aβ) clearance (150). What is unclear, however, is if the abnormal Treg response in the CNS and meninges is mediated by CCR7-dependent migration of T cells or if it is influenced by CCR7 activity of other cell types within the CNS, as the authors utilize a global CCR7 -/- in several experiments. Nonetheless, the authors and others have demonstrated that inhibiting Treg functionality in aged mice appears to improve some aspects of cognitive function in aged mice (10, 150). In agreement with this, the ability of peripheral lymphatics vessels to drain Tregs has also been demonstrated in skin inflammation models, where the failure to drain CCR7-KO Tregs at the site of inflammation to the lymph nodes resulted in increased local immune suppression (151).

Meningeal lymphatic drainage of T cells could also influence other diseases like brain cancer. Evidence from studies in periphery immunity demonstrates that blocking the ability of T cells to egress via afferent lymphatic vessels may actually improve the ability to fight some tumors (149). Here, the authors indicate that eliminating CXCL12 expression on afferent lymphatics or blocking CXCR4 on T cells led to retention of T cells at the tumor site and better tumor control. Finally, while it is tempting to speculate if meningeal lymphatic drainage of T cells may play a role in autoimmune diseases like EAE or MS, it should be noted that after high affinity antigen recognition, T cells typically do not have a sufficient exit program to leave their tissue environment (152). Merlini et al. convincingly demonstrated this phenomenon in the CNS and showed that when effector T cells are injected into subarachnoid space in EAE models these T cells rarely, if ever, migrate through the dural lymphatics system (27).

#### 3.3.4 Modifying phenotype of T cells at meningeal lymphatics

Similar to CNS-egressing DCs, the phenotypes of T cells that migrate from the CNS could have impacts on peripheral immunity. During EAE, our lab has identified large pools of T cells which are in close association to lymphatic vessels at the cribriform plate. Importantly, it was determined that during inflammation tolerogenic molecules like PD-L1 are expressed within cribriform plate lymphatic endothelial cells (**Figure 1B**) (37). MHC II was also found to be upregulated on inflamed cribriform LECs, with *in vitro* assays demonstrating that LECs had the ability to stimulate naive myelin specific T cells, and concurrently induce cell death through PD-L1 costimulation. Interestingly, blocking PD-L1 on cribriform plate LECs reduced this lymphatic induced T-cell death (37). Together this data suggests that the lymphatics vessels themselves can influence T cells in this niche which could impact both local and downstream immunity. While still early in our understanding of lymphatic endothelial cell induced modifications of immune cells, this data is supported by recent investigations into the peripheral lymph nodes which show that PD-L1 expressed on LECs can have important inhibitory properties on effector T cells (153, 154). Furthermore PD-1 stimulation on Treg populations by PD-L1 can support the survival and maintenance of Tregs in local tissue environments (155). Still an emerging concept, the ability of meningeal lymphatic vessels to locally regulate T cells offers interesting avenues of potential therapeutic intervention.

### 3.4 Monocytes and peripherally derived macrophages

Much like during peripheral inflammation, monocytes can differentiate into a variety of cell states, including monocytic dendritic cells (moDCs) within the brain tissue after infiltration. Depending on local signaling within the inflamed brain, these monocytes can transform into dendritic-like and activated macrophage cell states, as well as resemble resident microglia phenotypes. With this in mind, it appears that CNS-infiltrating monocytes can mirror many of the same anatomical migration pathways out of the brain as CNS-infiltrating dendritic cells outlined earlier. For example, Kaminski et al. demonstrated that GFP+ monocytes injected into the entorhinal cortex lesions migrate to the cervical lymph nodes in a time dependent manner, peaking in lymph nodes at around 7 days post injection. Within the brain, these injected monocytes appeared to move toward the olfactory bulbs, and after 48 hours were found aggregating along olfactory nerves, after which they likely utilized the nasal lymphatic system to reach cervical lymph nodes (**Figure 1B**) (60). Importantly, these monocytes appeared to adopt a “ramified” structure post-injection, which highlights the potential of monocytes to adopt dendritic/microglial-like or moDC phenotype once within the brain’s microenvironment. How moDCs reach cervical lymph nodes is still an ongoing debate and the receptors utilized by these moDCs are not completely understood, but could echo dendritic cells and use a similar CXCR4/CCR7 mediated mechanism of exit. However, both CNS and peripheral studies of moDCs indicate they often lack or express lower amounts of CCR7 which complicates understanding of their lymph node trafficking ability (156–158). Furthermore, moDCs are functionally distinct in their immunological functions from cDCs. For example, the antigen stimulating ability of moDCs has been documented to be significantly lower than cDCs, instead their role is primarily to migrate to and deliver antigen to resident lymph node cDCs which carry out the majority of priming interactions in the lymph node (159, 160).

Alternative to traditional lymph node trafficking pathways, a model of nasal inflammation recently showed that antigen-loaded monocytes lacking CCR7 can utilize the CCR5-CCL5 signaling to reach lymph nodes (161). Here, the authors report that a CCL5 gradient created from CCR7+ migratory DCs can guide CCR5+ monocytes through the lymphatic system. Whether or not the CCR5-CCL5 signaling axis also plays a role in DC-mediated monocyte trafficking out of the CNS and meninges remains to be shown, but if shown to occur, it could be a mechanism that CNS-draining DCs utilize to amplify antigen drainage from the CNS through the “shepherding” of local monocyte populations. A promising feature of this relationship is that it is shown to occur via the intranasal lymphatic system, which could have direct implications of CNS draining DCs and monocytes exiting through the cribriform plate. Most intriguing, the authors of this paper report that the draining monocytes expressed IL-10 and TGF-β, and had immunosuppressive characteristics once in the lymph node (161). As a result, identification of similar mechanisms occurring within the brain and around CNS drainage sites could prove essential in fully understanding how CNS-draining immune cell populations like monocytes and dendritic cells interact and potentially magnify specific responses to antigen. This is especially important as recent characterizations of channels between the skull bone marrow and dura, which appear to supply meningeal niches with a pool of monocytes in close association to meningeal lymphatic structures (83).

### 3.5 B cells

B cells play central roles in the maintenance of immune homeostasis. Through the past decades, increasing attention has been placed on the contribution of B lymphocytes to the pathogenesis of CNS diseases as abnormal B cell function can lead to both autoimmune and neurodegenerative diseases in the CNS. In EAE and MS, for example, B cells have been demonstrated to have opposite functions: contributing to both pathogenic progression and regulation of the disease in both species (162–167). While only few B lymphocytes can be observed in the lesions during the exceptionally early stages of MS, Henderson et al. found that in early stages of MS numerous B cells are present in the recently demyelinated tissues of newly forming CNS lesions (168). The study demonstrates that within these freshly demyelinated areas, a great number of B cells are present in the large perivascular spaces, in which B lymphocytes can account for as much as 90% of cells in certain cuffs. Machado-Santos et al. similarly showed that most CNS-infiltrating B cells are restricted to the perivascular space, surrounding only a singular or small number of larger veins at the lesion center. In addition, the paper also demonstrates that the quantity of B cells can exceed the number of T lymphocytes (even up to 4-times) in certain MS lesions (169).

Considerably less information is available about the drainage of B lymphocytes from the brain and regarding the kinetics and extent of B cell efflux during MS and EAE. Recently, Stern et al. investigated the B cell antibody repertoire present in the CNS and compared it to the B cell repertoire of the draining cervical lymph nodes in MS patients (170). The study reveals that the B lymphocytes populating the CNS lesions are related to the B cells of further CNS compartments including the choroid plexus and the pia mater, as well as to the B cells present in the draining CLNs. In addition, the paper demonstrates that both clonally expanded B lymphocytes and their less mature ancestors can be observed both in the CLNs and in the CNS. These findings provide evidence of the existence of a somewhat shared B cell repertoire between the CNS and the CLNs. Based on these data, the authors propose a model in which B cell trafficking can take place freely through the blood-brain barrier, and in a bidirectional manner between the CNS and the draining CLNs (170). A study by von Büdingen et al. identified clonally related B lymphocytes between the peripheral blood and the cerebrospinal fluid compartments of MS patients, which further points to the presence of an overlapping B cell antibody repertoire across the BBB. Notably, the paper not only demonstrates a robust and direct bidirectional exchange of B lymphocytes through the BBB, but also proposes that most of the closely related B cells that span different compartments across the BBB originate in the CNS (171).

With regard to meningeal lymphatics, Louveau et al., first reported the presence of B220+CD11c- B cells within dural meningeal lymphatic vessels (24). The migratory route of B cells from the CNS was very recently investigated by Brioschi et al., where they demonstrate that B lymphocytes populating the meninges can indeed migrate via the dural lymphatic vessels in the transverse and sagittal sinuses toward the draining CLNs (64). To determine this, researchers injected +CD19-tdTomato splenocytes into the CSF via the cisterna magna (ICM). 24 hours later, populations of B-cells could be observed in the B-cell zones of the draining CLNs. Additionally, the authors of this paper characterize dural to bone marrow skull channels that are hypothesized to supply the meningeal lymphatics with an extravascular B cell pool (**Figure 2**) (64). Whether or not lymphatic vessels present at the cribriform plate can also serve as drainage routes facilitating the efflux of B cells from the CNS compartments to the draining CLNs remains to be seen, but initial characterizations revealed only moderate levels of B cell-lymphatic interactions at the cribriform plate during EAE (37). Finally, what is less understood is the pathological implications of disrupted B cell migration from the CNS and meninges. Similar to what Da Mesquita et al. observed with T cell drainage, the proper homeostatic clearance or migration of B cells through meningeal lymphatics could influence the local meningeal immune response and aggregation of certain subtypes could shift the balance toward pathology (10). For example, B cells have been documented to form pathogenic ectopic lymphoid tissue aggregates in the meninges during diseases like multiple sclerosis (172). While these B cell aggregates in the meninges are still not fully understood, as APCs themselves, B cells have been shown to interact with T cells in the meningeal environment and could be major drivers of the early autoimmune disease process through the establishment pro-inflammatory sites at the brains borders (172). Thus, targeting the proper clearance and management of B cells aggregates during neuroinflammation may hold therapeutic promise, but more work is needed to understand this process and what role CNS-associated lymphatics play. Indeed, B cell depleting antibodies have already emerged as key therapeutic targets to treat autoimmune diseases like multiple sclerosis (173, 174), so alternative mechanisms to control B cell niches is the brain may yield similar therapeutic responses. Another proposal is that meningeal B cells can help influence and promote lymphangiogenesis regions and DC migration at CNS-associated lymphatic as seen in the periphery (175), interestingly human B cells in the CSF MS patients have reportedly elevated lymphangiogenic markers (176), but more work is needed to fully understand their role at CNS-draining lymphatics.

### 3.6 Neutrophils

Neutrophils have a wide repertoire of functions which include phagocytizing microbes, enhancing the recruitment of local immune cell populations, and regulating cytokine/growth factor secretion at inflammatory sites (177). Neutrophils are typically the first responders to the site of inflammation and are generally classified as short-lived cells with a high turnover rate. Most neutrophils that arrive at the tissue site ultimately die after completing effector duties. However, recent evidence suggests some neutrophils possess the ability to adopt a longer, multi-day lifespan upon exposure to inflammatory cytokines (178). In the context of neuroinflammation, neutrophils have been demonstrated to infiltrate CNS tissue and influence a variety of conditions from ischemic stroke (179), to MS/EAE (180), and to traumatic brain injury (181). During EAE, they appear to aggregate within meningeal spaces and within the parenchyma during onset to peak pathology, but appear to decline substantially during the more chronic phases. Additionally, large pools of neutrophils are present in the skull bone marrow directly adjacent to the CNS, and increase substantially during neuroinflammatory diseases like MS (148) and potentially egress directly from the bone marrow into the meningeal spaces via recently characterized skull channels (182–184) or across traditional blood brain barrier sites (185).

While neutrophil infiltration into the CNS is well established, neutrophil exit from the brain has not thoroughly been investigated. In peripheral skin models of inflammation, CCR7 has been shown to play a role in lymphatic clearance of aged neutrophils from active sites of inflammation (186). Additionally, Mac-1 and CXCR4 have been implicated as contributors to afferent lymphatic trafficking of neutrophils in peripheral inflammation models (187, 188). Recent data also suggests that many of the neutrophils leaving sites of inflammation may not be migrating toward lymphatic structures in all contexts, but instead re-entering the bloodstream. Using a murine sterile inflammation model, Wang et al. recently described that a subpopulation of tissue infiltrating neutrophils can utilize CXCR4 to transmigrate away from the injury site into the blood and return to bone marrow for pre-programmed apoptosis (189). A recent report utilizing two-photon intravital microscopy to look at LPS induced neuroinflammation described the phenomenon of “reverse transendothelial migration” of neutrophils from the CNS parenchyma back into the bloodstream (65). While the authors here report that neutrophil exit from the brain into the blood is relatively rare, similar observations in peripheral inflammation suggest that these reverse migrating neutrophils could have their own unique phenotype distinct from the tissue remaining neutrophils, namely a phenotype that avoids apoptosis at the site of inflammation (190). Additionally, the migration patterns of these CNS-emigrating neutrophils once entering the bloodstream is still inconclusive. Whether bone-marrow homing neutrophils exist within the brain remains to be investigated, but neutrophilic expansion within skull bone marrow is common in neuroinflammatory states like EAE/MS (148), so it is worth speculating that a proportion of this neutrophil increase in the skull bone marrow could be due to the recruitment of a subpopulation of neutrophils back into the marrow spaces rather than full myelopoietic expansion. In summary, neutrophil egress from sites of inflammation in the brain is still controversial, and more work is needed to understand if neutrophil migration from the brain plays a role, if any, in neuroinflammation.

## 4 Conclusion and future directions

During neuroinflammation there is a significant accumulation of immune cells in the CNS which can influence disease. Most of these recruited inflammatory cells in the CNS and meninges do not migrate out. However, subpopulations of DCs, T cells, B cells and to a lesser extent other myeloid cell populations appear to be licensed by chemokine receptors to exit the CNS through the meningeal lymphatics. These immune cells can then act as messengers from the CNS to secondary lymphoid organs to coordinate immune responses. Additionally, several studies have indicated that improper migration of these immune cells can enhance neuropathology. Our growing understanding of immune cell egress into afferent lymphatic vessels in peripheral models, leaves open the possibility that subtypes of other immune cells may engage in migration out of the meninges. Important questions for the future will be the distinction between true lymphatic mediated immune cell migration through receptor interactions (CCR7, CXCR4, etc) and passive clearance of immune cells from the CSF compartments, as well as the full implications of these CNS emigrating immune cells on neuropathology

For dendritic cells the evidence is most clear, but currently much of the evidence regarding immune cell migration from the CNS utilizes animal studies to track injected immune cells into the brain or CSF to the periphery. While this work has been instrumental in our understanding of the mechanics of immune cell migration from the CNS, this methodology can disrupt the very barriers which we seek to investigate and could provide false outflow pathways. Techniques to investigate and measure immune cell behavior in meningeal layers while preserving the integrity of the CNS’s barriers will be essential to fully elucidate preferred pathways of migration across a variety of disease states. New *in vivo* cell tracking methods in humans and animals will likely be key to delineate the full range of migration routes, as well as how aging and disease can influence these routes. Furthermore, investigations into fluid and small molecule drainage pathways from the CNS and meninges currently dominate the literature, outnumbering investigation into cell egress pathways. Cells, antigen, and fluid may utilize overlapping pathways in some cases to exit the CNS, but due to obvious size differences and the ability of immune cells to utilize chemotaxis, significant deviations are likely. Additionally, more human studies are needed to compare these cell migration pathways with the routes observed in animals. Unfortunately, live imaging techniques for humans often lack the resolution necessary to track fluid and cellular pathways, but recent non-invasive MRI techniques hold considerable promise and have recapitulated some of the findings from animal models regarding potential lymphatic-nerve fluid pathways (43). Cell labeling for MRI or PET imaging of immune cells presents several challenges in humans regarding timing, toxicity, and specificity but could ultimately allow for greater understanding of both CNS infiltration and drainage of immune cells (191, 192). Ultimately, basic reassessment of human CNS tissue samples with knowledge acquired from animal models could also yield similarly important results, as demonstrated by characterization of skull channels (183) and cranial nerve associated lymphatic cells (42) in humans.

A significant portion of our understanding of mechanisms of immune cell migration out of the CNS is derived from earlier investigations into peripheral tissues like the skin and lung. So, while it appears there is significant overlap in the receptors and chemokines used to ultimately exit CNS compartments as observed in peripheral organs, the unique cell types and anatomical boundaries of the CNS may require novel molecular pathways and interactions still undiscovered. Our understanding of immune cell migration from the CNS is ever growing in lock-step as the anatomical resolution of the various meningeal layers across the CNS increases. Reinvigorated interest and characterizations of meningeal lymphatic structures and the immune cells that populate them have provided new theories of CNS immune surveillance and the regulation of neuroinflammatory disease. Furthermore, several recent publications have reignited an ongoing debate regarding full contribution of dural lymphatic drainage during CNS autoimmune disease (27, 128), providing evidence that significant work is needed to clarify the role each CNS-associated lymphatic region provides toward overall CNS immunity. Nonetheless while murine data from our lab and others have heavily implicated the cribriform plate as a major migratory pathway for immune cells, particularly dendritic cells, it is apparent the other routes also contribute to immune cell egress and could play more prominent roles in humans such as those found along the dural lymphatics or other cranial nerves. Emerging evidence also indicates that afferent lymphatic vessels can not only locally regulate immune homeostasis through facilitating immune cells toward the lymph nodes, but can actually alter the phenotype of immune cells through direct interactions. New therapeutic strategies which target these lymphatic to leukocyte interactions could be instrumental in the treatment of CNS diseases. Several lymphatic - leukocyte pathways have now been investigated in both peripheral and CNS lymphatic systems (**Table 2**), but more work is needed to understand their full therapeutic potential across different homeostatic and neuropathological contexts.

**Table 2:** Potential targets of leukocyte–lymphatic interaction.

Immune Cell	Immune Cell Interactor	Lymphatic Vessel Interactor	Purpose/Notes	Demonstrated interaction in	
				Peripheral lymphatics	CNS-associated lymphatics
**Dendritic Cells**	**CCR7**	**CCL21/19**	Chemoattractant: migration/recruitment	193 194 195	14 36
	**CXCR4**	**CXCL12**	Chemoattractant: migration/recruitment	105 196	—
	**CX3CR1**	**CX3CL1**	DC transmigration	197	—
	**CLEC2**	**PDPN**	LEC adhesion, motility	198	—
	**Hyaluronan (hyaluronic** **acid, HA**	**Lyve-1**	LEC adhesion, DC transmigration	199	—
	**Integrin β1**	**VCAM-1**	LEC adhesion, DC transmigration	200 201	37
	**Mac-1**	**ICAM-1**	LEC adhesion, DC transmigration	200 202	—
	**CD137L**	**CD137**	Promotes CCL21-driven DC migration	203	—
	**VEGF-C**	**VEGFR-3**	Lymphangiogenesis	204	36
	**()?**	**Clever-1**	Transmigration, tolerance	205	—
	**Plexin A1 - NRP1 complex**	**SEMA3A**	LEC adhesion, DC transmigration	206	—
	**CD31^†^**	**CD31**	LEC adhesion, DC transmigration	196	—
	**CD99^†^**	**CD99**	LEC adhesion, DC transmigration	196	—
	**CD171/L1CAM^†^**	**CD171/L1CAM**	LEC adhesion, DC transmigration	207	—
**Macrophages** **/Monocytes**	**VEGF-C, -D, and -A**	**VEGFR-2, -3**	Lymphangiogenesis	208	36
	**CCR2**	**CCL2**	Lymphangiogenesis - Recruitment of VEGF expressing Macrophages/Monocytes	209	36 37
**T Cells**	**CCR7**	**CCL21/19**	Chemoattractant migration/recruitment	210	10 14
	**CCR10**	**CCL27**	Chemoattractant migration/recruitment	211	—
	**LFA-1**	**ICAM-1**	LEC adhesion, T cell transmigration	212	—
	**PD-1**	**PD-L1**	Immune Checkpoint/Tolerance	154, 213	37
	**TCR**	**MHC-I**	Priming, tolerance	214	—
	**TCR**	**MHC-II**	Priming. tolerance	215	37
	**IDO induced reprogramming**	**IDO**	Tolerance	216	—
	**iNOS induced reprogramming**	**iNOS**	Immunosuppressive	217	—
	**()?**	**Clever-1**	LEC adhesion, T cell transmigration	218	—
	**IFN-γ (Th1)**	**IFN-γ Receptor**	Suppression of lymphatic vessel growth ^A,B^ Upregulation of PD-L1 ^C^, IDO ^D^, iNOS ^E^	219 ^A^ 220 **^B^** 216 **^D^** 217 **^E^**	37 ^C^
**B Cells**	**VEGF-A**	**VEGFR-2**	Lymphangiogenesis	221 175	—
**Neutrophils**	**CXCR4**	**CXCL12**	Chemoattractant migration/recruitment	188	—
	**CCR7**	**CCL21/19**	Chemoattractant migration/recruitment	186	—
	**VEGF-D**	**VEGFR-2/3**	Lymphangiogenesis	222	—

List of some investigated targets of immune cell – lymphatic interactions, their function, and references organized according to anatomical region of investigation (Peripheral vs CNS lymphatics). Note this is not an exhaustive list, only potential targets as direct investigations into CNS lymphatic-leukocyte interactions are limited. Symbols: "—", interaction has no reference demonstrating evidence at this region. "?", binding site is not fully understood; proposed homophilic leukocyte – lymphatic interactions may also have heterophilic binding interactions, additional work is needed to fully map all binding interactions on leukocytes. Uppercase letters signify specific reference which applies to purpose of interaction.

## Author contributions

CL, CB, KK, wrote the manuscript. CL and CB made the figures and tables. ZF and MS edited and assisted with conceptual design. All authors reviewed the manuscript and agreed with regard to the contents. All authors contributed to the article and approved the submitted version.

## Glossary

**Table:**

CSF	Cerebral Spinal Fluid
dCLNs	Deep Cervical Lymph Nodes
DCs	Dendritic cells
EAE	Experimental autoimmune encephalomyelitis
IFN-γ	Interferon gamma
ISF	Interstitial Fluid
I.C	Intracerebral
I.C.M	Intracisterna magna
I.C.V	Intracerebroventricular
LECs	Lymphatic Endothelial Cells
LFA-1	Lymphocyte function-associated antigen 1
LPS	Lipopolysaccharide
LYVE1	Lymphatic vessel endothelial hyaluronan receptor 1
MHC I	Major histocompatibility complex class I
MHC II	Major histocompatibility complex class II
MS	Multiple Sclerosis
PD-1	Programmed cell death protein 1
PD-L1	Programmed death ligand 1
RMS	Rostral Migratory Stream
S1P	Sphingosine-1-phosphate
S1PR	Sphingosine-1-phosphate receptor
sCLNs	Superior Cervical Lymph Nodes
TGF-β	Transforming growth factor beta
VCAM-1	Vascular Cell Adhesion Molecule 1
VEGFR3	Vascular endothelial growth factor receptor 3
VEGF-C	Vascular endothelial growth factor C
